# Inflammaging and Cancer: A Challenge for the Mediterranean Diet

**DOI:** 10.3390/nu7042589

**Published:** 2015-04-09

**Authors:** Rita Ostan, Catia Lanzarini, Elisa Pini, Maria Scurti, Dario Vianello, Claudia Bertarelli, Cristina Fabbri, Massimo Izzi, Giustina Palmas, Fiammetta Biondi, Morena Martucci, Elena Bellavista, Stefano Salvioli, Miriam Capri, Claudio Franceschi, Aurelia Santoro

**Affiliations:** 1 Department of Experimental, Diagnostic and Specialty Medicine (DIMES), University of Bologna, Via San Giacomo 12, 40126 Bologna, Italy; E-Mails: rita.ostan3@unibo.it (R.O.); catia.lanzarini2@unibo.it (C.L.); elisa.pini5@unibo.it (E.P.); maria.scurti@unibo.it (M.S.); dario.vianello@unibo.it (D.V.); claudia.bertarelli@unibo.it (C.B.); cristina.fabbri12@unibo.it (C.F.); morena.martucci3@unibo.it (M.M.); elena.bellavista2@unibo.it (E.B.); stefano.salvioli@unibo.it (S.S.); miriam.capri@unibo.it (M.C.); claudio.franceschi@unibo.it (C.F.); 2 Interdepartmental Centre “L. Galvani” (CIG) University of Bologna, Via San Giacomo 12, 40126 Bologna, Italy; E-Mails: massimo.izzi@unibo.it (M.I.); mariagiustina.palmas@unibo.it (G.P.); fiammetta.biondi2@unibo.it (F.B.); 3 IRCCS, Institute of Neurological Sciences, Via Altura 3, 40139 Bologna, Italy; 4 National Research Council of Italy, CNR, Institute for Organic Synthesis and Photoreactivity (ISOF), Via P. Gobetti 101, 40129 Bologna, Italy

**Keywords:** aging, inflammation, inflammaging, cancer, mediterranean diet, nutrients, microRNAs, NU-AGE project

## Abstract

Aging is considered the major risk factor for cancer, one of the most important mortality causes in the western world. Inflammaging, a state of chronic, low-level systemic inflammation, is a pervasive feature of human aging. Chronic inflammation increases cancer risk and affects all cancer stages, triggering the initial genetic mutation or epigenetic mechanism, promoting cancer initiation, progression and metastatic diffusion. Thus, inflammaging is a strong candidate to connect age and cancer. A corollary of this hypothesis is that interventions aiming to decrease inflammaging should protect against cancer, as well as most/all age-related diseases. Epidemiological data are concordant in suggesting that the Mediterranean Diet (MD) decreases the risk of a variety of cancers but the underpinning mechanism(s) is (are) still unclear. Here we review data indicating that the MD (as a whole diet or single bioactive nutrients typical of the MD) modulates multiple interconnected processes involved in carcinogenesis and inflammatory response such as free radical production, NF-κB activation and expression of inflammatory mediators, and the eicosanoids pathway. Particular attention is devoted to the capability of MD to affect the balance between pro- and anti-inflammaging as well as to emerging topics such as maintenance of gut microbiota (GM) homeostasis and epigenetic modulation of oncogenesis through specific microRNAs.

## 1. Inflammaging as a Major Component of Aging, Age-Related Diseases and Cancer

Human aging is a complex, extremely heterogeneous and dynamic trait determined by a number of environmental, genetic, epigenetic, and stochastic factors [1]. A pervasive feature of human aging and probably one of its major causes, is represented by the chronic, low-level state of systemic and sterile (in the absence of overt infection) inflammation called “inflammaging” [2,3]. Indeed, inflammation has been recently included among the seven pillars of aging [4]. It can be beneficial as an acute, transient immune response to harmful conditions, facilitating the repair, turnover and adaptation of many tissues. However, during aging, inflammatory response tends to become chronic and of low grade, leading to tissue degeneration.

Indeed, inflammaging is characterized by a general increase in plasma levels and cell capability to produce pro-inflammatory cytokines such as Interleukin-6 (IL-6), Interleukin-1 (IL-1) and Tumour Necrosis Factor-α (TNF-α) and by a subsequent increase of the main inflammatory markers, such as C-reactive protein (CRP) and serum amyloid A (A-SAA) [2,5]. This generalized pro-inflammatory status, interacting with the genetic background and environmental factors, potentially triggers the onset of the most important age-related diseases, such as cardiovascular diseases, atherosclerosis, metabolic syndrome, type 2 diabetes, obesity, neurodegeneration, arthrosis and arthritis, osteoporosis and osteoarthritis, sarcopenia, major depression, frailty and cancer [6,7].

The hypothesis of a possible correlation between cancer and inflammation was firstly formulated by the Greek physician Galenus [8,9]. In 1863 Rudolph Virchow, a pioneer of cellular pathology, noted inflammatory cells within tumor mass and that tumors arise at sites of chronic inflammation [10,11]. A functional framework developed by Hanahan and Weinberg (2000) characterizes cancer by six biological hallmarks, able to regulate the conversion of normal cells in cancer cells: self-sufficiency in growth signals, insensitivity to growth inhibitory signals, limitless replicative potential, the ability to evade programmed cell-death (apoptosis), the ability to sustain angiogenesis, the ability to invade tissues and metastasize [12]. Studies have also supported the important role of inflammatory cells and cytokines in the tumor microenvironment [13,14,15]. In 2011, Weinberg and Hanahan proposed four additional new cancer hallmarks: ability to evade the immune system, presence of inflammation, tendency towards genomic instability and dysregulated metabolism [16]. The correlation between chronic inflammation and cancer has been supported by epidemiological and experimental studies on humans and animal models [13,15,17] along with the observation that preventive treatments with anti-inflammatory drugs such as aspirin or cyclooxygenase-2 (COX-2) inhibitors reduce the risk of developing colorectal and breast cancer and even mortality [15,18,19]. Chronic inflammation affects all cancer stages, increasing the onset risk, supporting the initial genetic mutation or epigenetic mechanism leading to cancer initiation [20,21,22], promoting tumor progression, and supporting metastatic diffusion [9,22,23,24,25].

We recently hypothesised that it is important to distinguish between systemic inflammaging and local inflammaging. While the pathological and pathogenetic role of circulating pro-inflammatory compounds and cytokines is unclear and possibly negligible (a marker of inflammation rather than an active player), there are a number of observations and papers suggesting that the local production of inflammatory cytokines can have strong deleterious effects, as we recently suggested in the case of breast cancer niche as a paradigmatic example [26,27]. Therefore, it is tempting to speculate that the important aspect to be considered in inflammaging and cancer is not the mere increase in inflammatory mediators but rather the source, and therefore the local targets, of these mediators. The tangled interplay among local immune responses and systemic inflammation and their influence on clinical outcomes in cancer has been recently reviewed [28].

Different types of tissues (muscle, adipose tissue), organs (brain and liver), systems (immune system) and ecosystems (gut microbiota, GM) may contribute to the systemic inflammatory state, through altered production of pro-inflammatory and/or anti-inflammatory mediators [5,7,26,29,30].

Inflammaging can be influenced by many other factors, such as microRNAs (miRs) and agalactosylated *N*-glycans, together with the products and metabolites of the intestinal microbiota.

Additionally, some mitochondrial components, including mtDNA and other “cellular debris” released outside of the cells, as a consequence of natural cell turnover/damage, could trigger and sustain a sort of “physiological inflammatory tone” that increases with age [31]. This conceptualization can be extended to nutrients, whereby an excess of nutrients could be therefore capable of triggering an inflammatory response [32,33,34] that has been dubbed “metaflammation” [35], contributing to the above-mentioned physiological inflammatory tone.

### Inflammatory Sources for Cancer Development

Apart from inflammaging, viruses, bacteria and parasite infections as well as the exposure to chemical or physical agents can support chronic inflammation and have been linked to several cancer types [36,37,38]. Similarly, unresolved inflammation unrelated to infections can also contribute to carcinogenesis as observed in Barrett’s metaplasia, chronic pancreatitis or esophagitis [21,38,39,40,41,42,43] or in autoimmune diseases [21].

Obesity plays a central role in carcinogenesis since adipose tissue has been recognized as an endocrine source of mediators (hormones, acute-phase proteins, cytokines, adipokines and growth factors, [44]) able to sustain a chronic low-grade inflammation. During the last fifteen years, obesity has been associated with several types of tumors such as breast, endometrium, prostate, kidney, esophagus, stomach, colon, pancreas, gallbladder, and liver [45,46,47,48,49] and also with an increased cancer aggressiveness, risk of relapse and mortality [49].

A large amount of data indicates that inflammation is closely connected to oxidative stress. Reactive oxygen species (ROS) are continuously produced by our cells as a by-product of oxidative metabolism and are essential for several physiological functions and signalling pathways. However, an excessive accumulation of ROS may cause cellular oxidative damage to nucleic acids and proteins in cells of several systems including the endocrine and the immune systems [26,50]. We have recently hypothesised that most of the deleterious effects of excessive oxidative stress in tissues and organs can be mediated by the induction of unwanted inflammatory reactions [50].

Indeed, an important characteristic of tumor promoters is their ability to recruit inflammatory cells and to stimulate them to generate ROS [51]. Mast cells and leukocytes recruited to the site of damage lead to a “respiratory burst” due to an increased uptake of oxygen and, thus, an increased release and accumulation of ROS at the site of damage [20]. On the other hand, inflammatory cells also produce soluble mediators, such as metabolites of arachidonic acid, CRP, cytokines (IL-1, IL-6), and chemokines, which act by further recruiting inflammatory cells to the site of damage and producing more reactive species. This sustained inflammatory/oxidative environment leads to a vicious cycle, which can affect healthy neighboring epithelial and stromal cells, by inducing DNA damage and activating epigenetic mechanisms, and over a long period of time may lead to carcinogenesis.

**Figure 1:**
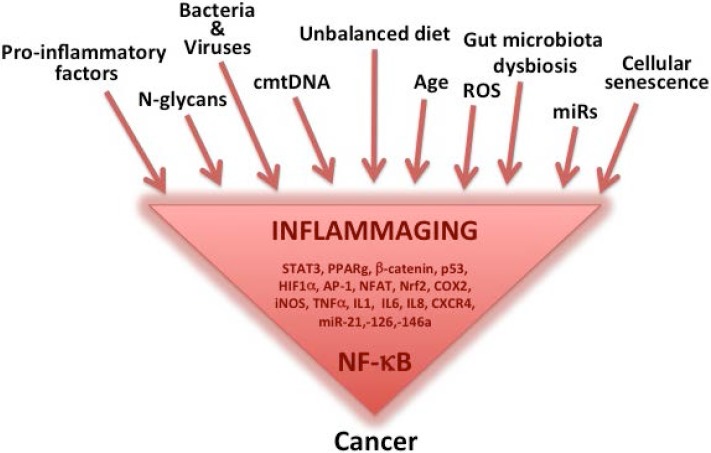
Among the main causes of inflammaging, we found the accumulation of pro-inflammatory factors, viruses and bacteria, age, reactive oxygen species (ROS) and cellular senescence. Inflammaging can also be influenced by many other factors, including non-immunological ones, and those not directly related to inflammation, such as microRNAs (miRs), circulating mitochondrial DNA (cmtDNA) and agalactosylated *N*-glycans, together with the products and related metabolites of the intestinal microbiota. Several pathways and molecules are triggered by these factors, which then are able to activate the nuclear transcription factor NF-κB which could be considered as a hub in carcinogenesis, linking inflammaging, cellular senescence and cancer.

During tumor progression, immune and inflammatory cells produce cytokines and chemokines, which facilitate cancer cell survival and proliferation, and promote the angiogenic switch enhancing tumor growth [52]. Cytokines and chemokines also induce further recruitment and differentiation of immune cells in the tumor microenvironment [53]. The key mediators can activate signal transduction cascades and induce changes in transcription factors, such as nuclear factor-κB (NF-κB), signal transducer and activator of transcription 3 (STAT3), PPAR-γ, β-catenin, p53, hypoxia-inducible factor-1α (HIF-1α), activator protein-1 (AP-1), nuclear factor of activated T cells (NFAT), and NF-E2 related factor-2 (Nrf2), which mediates immediate cellular stress responses [54]. All these molecules are regulated by the transcription factor NF-κB [55], which could be considered as a “hub” in tumorigenesis linking cellular senescence, inflammaging and cancer [23]. As summarized in Figure 1, almost all gene products involved in inflammation are indeed regulated by the activation of NF-κB (e.g., TNF-α, IL-1, IL-6, chemokines, COX-2, 5LOX, CRP) [56] and NF-κB is activated in response to several well known cancer risk factors such as smoke, stress, dietary agents, obesity, infectious agents and irradiation. Moreover, NF-κB has been associated with transformation of cells [57] and is constitutively active in most tumor cells. Cellular senescence, a tumour suppressive stress response, is associated with a secretory phenotype that might be an important additional contributor to chronic inflammation [58]. Senescent cells are in fact characterized by the capability to produce high amounts of pro-inflammatory proteins [59,60]. The senescent phenotype is also accompanied by an upregulation of the DNA damage-response system and recently it has been proposed that the accrual of DNA damage with age can contribute significantly to inflammaging via the production of IL-6 [27]. In the cancer field this phenomenon, related to the propagation to bystander cells of DNA damage, DNA damage response and inflammation, has been conceptualized as “para-flammation” [61].

All these exogenous and endogenous danger signals (viruses, bacteria, including the GM and its products, damaged and senescent cells, cell debris, altered/modified proteins, *N*-glycans, mtDNA, ROS, *etc.*) are overall conceptualized by our group as “garbage”, able to trigger inflammaging and inflammation. Indeed, all these “dysfunctional” molecules can be sensed by receptors of the innate immune response and thus are potential stimulants of pro-inflammatory responses. This “garbage” is an inevitable byproduct of the normal metabolism, but its accumulation becomes evident with advancing age and/or in pathological conditions [3], due to the lifelong exposure to exogenous/endogenous insults on one side, and to the decreased capacity of the ubiquitin-proteasome system [62,63] and autophagy [64] to cope with these products on the other side.

## 2. The Mediterranean Diet

### 2.1. The Mediterranean Diet: Definitions and Characteristics

In literature, there are various definitions of the Mediterranean Diet (MD) but they generally share the main components: a high consumption of vegetables, fruits, whole grains, legumes, olive oil and fish (especially marine species), a low intake of saturated fats such as butter and other animal fats, red meat, poultry, dairy products and a regular but moderate consume of ethanol mainly consisting of red wine during meals. Some of these features overlap with other healthy dietary patterns, whereas other aspects are unique to the MD.

The MD is the typical dietary pattern of the populations bordering the Mediterranean area. The traditional MD has been accepted and acknowledged by the scientific community following the publication by Ancel Keys and colleagues showing results from the Seven Countries Study. The purpose of this longitudinal epidemiological study, started in the late 1950s, was to examine the relationships between lifestyle and dietary factors and cardiovascular diseases in populations from different regions of the world (the USA, Northern Europe, Southern Europe and Japan). Resulting data indicated that the mortality rate for coronary heart disease was higher in the USA and Northern Europe in comparison to Southern Europe. In particular, subjects from Greece and Italy showed the lowest mortality for cardiovascular diseases [65]. In 2003, the PREDIMED study found that the MD supplemented with extra virgin olive oil or tree nuts was able to prevent cardiovascular diseases in comparison to a low-fat diet [66].

Between 2005 and 2010, the Moli-sani study showed that higher adherence to the MD was associated with a reduction of leukocytes and platelets suggesting that the set of foods composing the MD could have an anti-inflammatory action and a protective effect on many diseases (primarily atherosclerosis) with an inflammatory pathogenesis [67]. Overall, these and other studies indicated the existence of inverse associations between MD and total mortality [68], the incidence of coronary heart disease [69,70], thrombotic stroke [71], and with the development of various forms of cancer [72,73].

**Figure 2:**
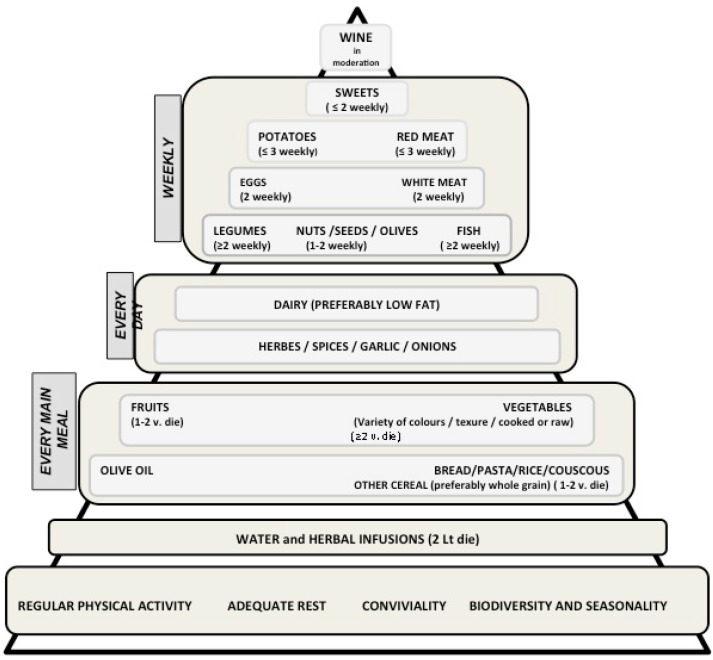
Pyramid of Mediterranean lifestyle (inspired by the “Mediterranean Diet Foundation” Barcelona Spain). The size of different sectors of the pyramid is directly proportional to the frequency of use of that particular food or food group. At the base of the pyramid there are healthy habits and groups of foods to be eaten daily and in large quantities (*i.e.*, fruit, vegetables, *etc.*). The upper levels show the foods to be eaten moderately (*i.e.*, sweets, red meat, *etc.*).

The international scientific community has accepted the role of the MD in increasing life expectancy and improving general health contributing to the spread of the MD pattern as a central pillar of programs and public health policy in many countries, from the USA to Europe. The MD is not only a diet but represents a lifestyle. The “Mediterranean Diet Foundation” developed a chart of the food pyramid, which includes information closely related to the Mediterranean lifestyle, cultural and social order as well as the importance of exercise and conviviality. Figure 2 highlights the importance of the Mediterranean lifestyle, including factors not associated with the use of particular foods.

The carbohydrate composition of the MD deserves special attention. Consumption of unrefined whole grain carbohydrates as a preferred choice has a double action: it limits the elevation of postprandial blood glucose and ensures a good supply of fiber. In fact, whole grain cereals have a lower glycemic index (GI) than refined products made with white flour, white rice and sugar. The consumption of low-GI foods avoids sudden increases in blood glucose, limits the secretion of insulin, and, therefore, inflammation [74]. Moreover, low-GI products have an anti-atherogenic action, decreasing the production of atherogenic lipoproteins, oxidized LDL and inflammatory markers [75]. The consumption of whole grains, legumes and other plant foods recommended by the MD brings a high amount of fiber (β-glucans, arabinoxylans, galactomannans, pectins) that increases satiety and helps to control weight. Numerous scientific results showed that dietary fiber promotes gut health and prevents cardiovascular disease, cancer, obesity and diabetes [76]. In the gut, prebiotic fiber (inulin, lactulose and galactooligosaccharides) can be selectively fermented by Bifidobacteria and/or Lactobacilli. The growth of these microorganisms maintains homeostasis and functionality of the intestinal microbiota and reduces the risk of dysbiosis [77]. Moreover, fiber is an effective “carrier” of bioactive antioxidants (vitamins C and E, carotenoids, and polyphenols).

The MD is characterized by a high content of “good fats”, monounsaturated (MUFA) and polyunsaturated (PUFA) fatty acids, present in marine fish, vegetable oils (especially olive oil), in nuts and seeds, and by a low intake of saturated fatty acids and hydrogenated oils (trans fats). In particular, the MD provides an optimal dietary fat profile characterized by a low intake of saturated and ω-6 fatty acids and a moderate intake of ω-3 fatty acids [78]. The ratio between ω-6 and ω-3 PUFAs plays an important role in the modulation of inflammation and blood coagulation [79] and is one of the most powerful anti-inflammatory features of this diet.

In terms of micronutrients, the MD is rich in B vitamins (B1, B2, niacin, B6, folate or B12), antioxidant vitamins (vitamins E and C) and minerals, especially iron, selenium, phosphorus and potassium.

Plant foods constitute the core of MD and are characterized by a high content of “non-nutritive” components (phytochemicals), including polyphenols, phytosterols and carotenoids. Phytochemicals are bioactive substances known to combat cellular inflammation due to their powerful antioxidant action. Data from PREDIMED and other studies suggested that the efficiency of the dietary antioxidants in modulating the plasma antioxidant capacity depends on the health status of individuals. In fact, the best results are obtained in people with some risk factors (for example, smokers) or cardiovascular disease or in subjects with a low initial plasma antioxidant capacity. The healthy subjects with low levels of oxidative stress showed a reduced responsiveness to antioxidants. In addition, some studies indicate a negative effect of antioxidant supplements in overall mortality and mortality caused by cardiovascular disease, diabetes and some types of cancer. This is certainly due to the complexity of the interactions between endogenous and exogenous antioxidants and to the existence of homestatic mechanisms of control intended to prevent an overload of reducing agents maintaining the physiological state of homeostasis [80,81]. Therefore, although an adequate intake of antioxidants is needed to counteract oxidative stress, these compounds should be introduced through plant foods such as fruits, vegetables, whole grains, nuts and seeds naturally present in a healthy and complete nutritional model such as the MD.

Diet scores are increasingly being employed to define MD adherence in epidemiological studies [82]. Trichopoulou and colleagues proposed in 1995, and subsequently updated, the Mediterranean Diet Score (MDS) [83]. This simple score was constructed by assigning a value of 0 or 1 for each of the nine components. Therefore, the total MDS ranges from 0 (minimal adherence to the traditional Mediterranean diet) to 9 (maximum adherence) [70]. The NIH-AARP Diet, the Health Study, the European Prospective Investigation into Cancer and Nutrition (EPIC) and other studies used the MDS and other scores later derived from it to confirm the correlation between adherence to MD and mortality reduction, demonstrating the MD protective role in regard to the prevention of cancer, cardiovascular and other chronic diseases [72,73,84,85].

### 2.2. The Preventive Role of the Mediterranean Diet on Cancer

#### 2.2.1. Epidemiological Studies

Nutrition represents an easily modifiable factor able to contrast inflammation and oxidative stress. Growing evidence indicates the beneficial and preventive role of the Mediterranean Diet (MD) in the onset of cancer and other diseases associated with increased level of inflammation, oxidative damage and angiogenesis. A recent metanalysis of all the observational studies regarding the adherence to MD in relation to cancer risk [86] showed that MD is associated with a significant reduction of overall risk of cancer incidence and mortality by 10%. In particular, increased adherence to the MD reduces the likelihood of having colorectal cancer CRC, even among obese and diabetic patients suggesting potential benefits of this dietary model on CRC risk factors [87,88,89]. Contrasting data are reported on other forms of neoplasms. While the metanalysis by Schwingshackl and Hoffmann indicates a reduction of the risk of prostate cancer by 4%, a recent paper reported that a higher Mediterranean Diet Score (MDS, see Section 2.1 for details) was not associated with risk of advanced prostate cancer or disease progression in a cohort of 47867 men prospectively followed for 24 years. However, in the same subjects, the adherence to the MD was associated with lower overall mortality after diagnosis of nonmetastatic prostate cancer [90]. Some studies investigating the role of the MD on oral and pharyngeal cancer reported an inverse association between the risk of this neoplasm and adherence to the MD, as measured by various indexes, and indicated a stronger effect in younger subjects [91,92,93]. A meta-analysis by Schwingshackl and Hoffmann did not seem to confirm an effect of the MD on the risk of breast cancer, even if a subgroup of case-control study showed that the risk of this cancer could be reduced by 18% in women adhering to the MD. In particular, a recent study on 500 Greek middle-aged women showed that one unit increase in MDS was associated with 9% lower risk of breast cancer. It is worth noting that the protective effect of the MD against breast cancer seemed to depend on individual’s characteristics and potential risk factors, *i.e.* obesity, physical activity, smoking, age at the menarche, menopausal status. In the above described cohort, the beneficial effect of MD on breast cancer risk is observed only in normal weight, non smoking women and in women who did not present an early menarche (<12 year old) [94]. The studies describing the MD preventive action against various types of cancer suggested that this healthful dietary pattern acts through several mechanisms, decreasing the dysregulated free radical production and inflammation [80,95,96].

#### 2.2.2. Chemoprotective Effects of Polyphenols on Inflammation and Cancer

The abundant consumption of fruit, vegetables, grains, legumes, olive oil and the moderate intake of red wine introduces, in the organism, high levels of different polyphenols and plant bioactive compounds that initially were known as antioxidants but later were studied for their anti-inflammatory, anti-tumor, anti-atherogenic abilities that could not be explained solely on the basis of their antioxidant properties. In fact, a series of investigations into the mechanism of action of these molecules have shed light on the fact that polyphenols do not merely exert their effects only as free radical scavengers, but may also modulate cellular signaling processes involved in inflammatory response or may themselves serve as signaling agents [97]. In particular, dietary polyphenols from olive oil (oleuropein, hydroxyltyrosol) and from red wine (resveratrol) were shown to modulate the eicosanoids pathway through the inhibition of cellular enzymes such as phospholipase A2 (PLA2), cyclooxygenase (COX-1 and COX-2) and lipoxigenase (LOX). This action reduces the cellular production of arachidonic acid and inflammatory prostaglandins and leukotrienes [98]. Other studies showed that also quercitin, the most abundant and widespread natural flavonoid present in a variety of fruit and vegetables, inhibited COX and LOX in different cellular animal models exerting an anti-inflammatory action [99,100,101]; quercitin is also able to rejuvenate senescent fibroblasts by activating proteasome function [102]. Olive oil and red wine polyphenols reduce inflammatory angiogenesis, a key pathogenic process in cancer and atherosclerosis, in human cultured endothelial cell through the inibithion of COX-2 protein expression, prostaglandin production and MMP-9 release. This effect is accompanied by a substantial reduction of ROS levels and NF-κB activation [103]. A variety of polyphenols (quercitin, apigenin, luteolin, kaempferol, myricetin) are able to modulate the inflammatory process through the inhibition of nitric oxide (NO) production by supressing nitric oxide synthase (NOS) enzyme expression and/or activity [104,105,106]. A plethora of studies on human and animal cellular models have shown that different flavonoids such as quercitin and phenolic compounds from extra virgin olive oil interfere with the expression, production and/or function of cytokines/chemokines such as TNF-α, IL-1β, IL-6, IL-8, MCP-1, IFN-γ and IL-10, contributing to the control of the balance between pro- and anti-inflammatory mediators and exerting a potent anti-inflammatory activity. These compounds, as natural antioxidants are able to efficiently modulate the redox status of cells and strictly regulate the inducible gene expression of inflammatory mediators [107,108,109,110,111,112]. Moreover, polyphenols are involved in multiple steps of the NF-κB activation process, which represent an important and very promising pathway for the treatment and prevention of inflammatory diseases and cancer [113]. A recent review described the action played by dietary polyphenols in the inhibition of cancer cell growth due to their ability to modulate the activity of multiple targets involved in carcinogenesis through simultaneous direct interaction or modulation of gene expression. In particular, polyphenols are able to reduce and prevent the cross-talk between ErbB receptors, NF-κB and the Hedgehog (HH)/glioma-associated oncogene (GLI) pathways representing three of the main signal transduction pathways for neoplastic transformation [114].

Phenolic compounds are able to modulate the pathways of mitogen-activated protein kinases (MAPKs). These specific transcription factors play a central role in cell growth, proliferation, death and differentiation by modulating gene transcription in response to changes in the cellular environment. MAPKs regulate the transcription and traslocation of inflammatory mediators and represent potential targets for new anti-inflammatory molecules. Kaempferol, chrysin, apigenin and luteolin inhibit the activity of the three mitogen activated protein kinases, ERK, JNK and p38, blocking TNF-α stimulated ICAM-1 expression in respiratory epithelial cells [115]. Even quercitin inhibits a wide range of pro-inflammatory genes through the regulation of the MAPK pathway. In particular, this compound inhibits ERK, JNK and their phosphorylated forms, suppressing the transcription and the production of TNF-α in human monocytes [116].

**Figure 3:**
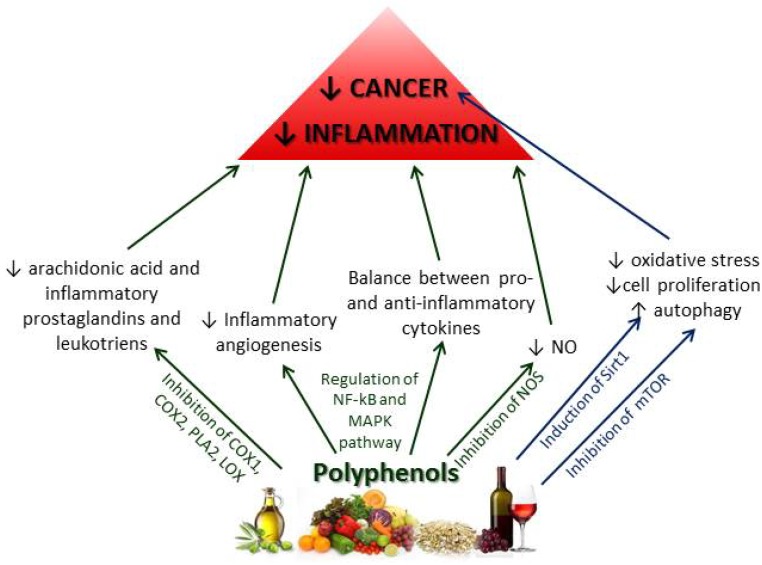
Polyphenols control and reduce inflammation through a series of pathways preventing cancer and other age-related diseases with an inflammatory pathogenesis. Moreover, resveratrol, quercitin and other polyphenols exerted their anti-cancer and chemopreventive action through mechanism that mimic caloric restriction (sirtuin and mTOR pathways).

In this context, quercitin and other dietary polyphenols typical in the MD, are able to reduce inflammation through a series of different but interconnected mechanisms, and may represent very attractive anti-inflammatory agents and safe non-pharmacological tools for the prevention and treatment of cancer (Figure 3). Moreover, polyphenols exerted their anti-cancer and chemopreventive action through the regulation of mTOR (mammalian target of rapamycin) and the sirtuins pathways by mechanisms that mimic caloric restriction [117]. In particular, quercitin is able to inhibit mTOR activity by multiple pathways. The signalling pathway of mTOR stimulates cell growth and proliferation inducing protein synthesis and inhibiting autophagy in case of food wealth. When essential cellular functions are endangered by insufficient nutrient supply, as in the case of caloric restriction, mTOR activity is blocked and cytosolic compounds are recruited for degradation and recycled by autophagy. On the contrary, the mTOR complex is often hyperactivated in cancer [118] and therefore is considered to be an interesting and attractive therapeutic target for anti-cancer therapy. Sirtuins role in cancer development is very complex and contradictory since different members of the sirtuin family are implicated in various cancer types. Several studies corroborate the possibility of the inhibitory effect of sirtuins on inflammation [119,120] by influencing mainly the NF-κB pathway [121,122] or TNF-α and IL-6 expression (SIRT6) [123,124]. A series of polyphenols have been shown to induce SIRTt1, defined as a guardian against oxidative stress and DNA damage [117], acting as tumor suppressor and attenuating cellular proliferation, but also speeding up tumorigenesis activating oncoproteins. Such dual functions of SIRT1 may be determined, at least in part, by its subcellular localization [125,126]. Data from mice indicated that a diet rich in olive oil polyphenols reduced oxidative stress, inducing NRF2 and the expression of its target genes coding for antioxidant enzymes, and increasing SIRT1 gene expression [127]. Systematic molecular analysis of olive oil phenolic extracts identified secoiridoids as a family of compounds with a strong anti-cancer activity related to the activation of anti-aging/cellular stress-like gene signatures, including endoplasmic reticulum (ER) stress and the unfolded protein response as well as SIRT1 and NRF2 signaling [128]. Several studies demonstrated that resveratrol is able to induce Sirt1 and clinical investigations indicated a role of this compound in the modulation of enzyme systems involved in carcinogen activation and detoxification, suggesting a possible mechanism by which resveratrol inhibits carcinogenesis. Unfortunately, phase II studies have failed to confirm the safety and efficacy of resveratrol in patients with relapsed/refractory multiple myeloma [129].

While many epidemiological studies have associated the consumption of polyphenols with a decreased risk of developing several diseases such as cancer, intervention studies have not always confirmed these effects. This discrepancy may in part depend on potential differences in doses, interactions with the food matrix, and differences in polyphenol bioavailability that limit their overall biological effectiveness. In addition to endogenous factors such as microbiota and digestive enzymes, the food matrix considerably affects bioaccessibility, uptake, and further metabolism of polyphenols. In particular, dietary fiber (such as hemicellulose), divalent minerals, and viscous and protein-rich meals are likely to cause detrimental effects on polyphenol bioaccessibility. In addition, certain food preparation techniques may alter nutrient composition and structure reducing polyphenol bioavailability [130].

#### 2.2.3. Other Mediterranean Diet Components and Their Effects on Inflammation and Cancer

Several epidemiological studies have linked the increased consumption of lycopene, the red and lipophilic carotenoid representing the most relevant functional component of tomatoes, with decreased prostate cancer risk. *In vitro*, several experiments showed that lycopene enhances the antioxidant response of prostate cells, inhibits proliferation, induces apoptosis and decreases the metastatic capacity of prostate cancer cells. However, the *in vivo* effectiveness of lycopene as a chemoprotective has still to be proven [131]. Several clinical trials have shown that carotenoids, vitamin A, and vitamin E based antioxidant supplements do not possess preventive effects on cancer and may be harmful and increase mortality especially in well-nourished populations. Therefore, the optimal source of antioxidants seems to come from diet, not from antioxidant supplements in pills or tablets [132].

The high presence of fiber typical of the MD is probably at the basis of the reduced incidence of colorectal cancer. Dietary fibers possess a proven anti-inflammatory action decreasing systemic inflammation-associated biomarkers such as CRP, IL-6, and TNF-α [133,134,135], as well as inhibiting COX-2 and iNOS activities and gene expression [136].

Fermentable dietary fibers shift the gut microbial populations by providing substrates for bacterial fermentation. In particular, fructooligosaccharides and galactooligosaccharides increase the fecal populations of *Bifidobacteria* and *Lactobacillus* [137] and these changes in gut flora population can result in a modulation of inflammatory processes [138]. Moreover, the consumption of fiber-rich foods avoids glycemic “spikes” and rapid insulin release that may adversely regulate multiple mechanisms, including (acutely and/or chronically) oxidative stress, inflammation, low-density lipoprotein oxidation, protein glycation, and blood coagulation [74]. Finally, dietary fibers favour an enlargement of the bulk of stool fasting intestinal transit and reducing the contact of potentially carcinogenic and toxic coumpounds with gastrointestinal epithelium [139].

Data from PREDIMED and other studies indicated an association between walnut consumption and reduced risk of cancer and mortality particularly in the context of the MD. Numerous components of walnuts, including α-linolenic acid (ALA), ellagitannins, γ-tocopherol, melatonin, β-sisterol and fiber may counter inflammation-related cancer mechanisms [140]. In particular, one-year of the MD supplemented with either extra virgin olive oil or mixed nuts (walnuts, almonds, and hazelnuts) *versus* a low fat diet decreased intercellular adhesion molecule-1 (ICAM-1), IL-6, TNFR60, and TNFR80 levels in adults [141].

The frequent consumption of marine fish typical in the MD provides a high quantity of ω-3 polyunsatured fatty acids. A high ω-3 to ω-6 fatty acids ratio has been associated with a reduced risk of cancer, especially breast cancer, and with improved prognosis [78,142]. ω-3 Fatty acids exert anti-angiogenic effects and have anti-inflammatory and immunosuppressive properties reducing inflammation through different mechanisms. In particular, EPA (eicosapentaenoic) and DHA (docosahexanoic) ω-3 fatty acids partially replace arachidonic acid as eicosanoid substrate in all cell membranes but especially in erythrocytes, neutrophils, monocytes and liver cells, thus suppressing the production of ω-6 pro-inflammatory eicosanoids. In addition, EPA and DHA suppress the NF-κB pathway and modulate plasma membrane micro-organization (lipid rafts), in particular relatively to the function of Toll-like receptors (TLRs), and T-lymphocyte signaling molecule recruitment to the immunological synapse [143].

The traditional MD is characterized by a low consumption of red and processed meat, which is often associated with an increased risk of colorectal cancer. Processed meat intake is linked to cancer risk through different mechanisms including the production of carcinogenic heterocyclic amines, polycyclic aromatic hydrocarbons and N-nitroso compounds as well as the high content of saturated fatty acids that enhances the prostaglandin system feeding the arachidonic acid and PGE_2_ pro-inflammatory pathways [144].

In contrast to the MD, the Western diet, characterized by a low intake of nutrient-rich food (fruit, vegetables, whole grain cereals, legumes and fish) and by an over consumption of salt, refined sugars, saturated fatty acids and a low ω-6:ω-3 fatty acids ratio, damages the immune system leading to an increased level of inflammation and increased onset of cancer [145].

### 2.3. The Mediterranean Diet Epigenetic Regulation: the Role of microRNAs

MiRs are small non-coding RNAs involved in the post-transcriptional regulation of gene expression and are recently recognized as diagnostic and prognostic biomarkers for many age-related diseases and aging [59,146].

MiRs play a critical role in basic biological processes such as cellular differentiation, apoptosis, cell proliferation, metabolism, inflammation, stem cells development, immune modulation and carcinogenesis [147,148]. It has also been shown that miR expression is tissue specific, altered with age, and can define the physiological context of the cell, including disease [149]. Alterations in the expression of specific miRs have also been reported to play a role in oxidative stress-induced inflammation [20,51,52,53,54,150]. Recently, our team identified three miRs we named “inflamma-miRs”: miR-21, -126 and -146a, which target mRNAs belonging to the NF-κB pathway [151].

Several patterns of expression of miRs were exclusive of certain tumors and reflect the differentiation state of tumor development [152]. Especially in the last 10 years, many studies have shown that dysregulation of miR expression underlies many human cancers, both as oncogenes (oncomirs) or tumor suppressors [153,154,155]. Therefore, the possibility of using miRs to block accumulation of senescent cells to inhibit the establishment of a microenvironment favoring cancer development and progression could be a potential new approach to cancer prevention. 

In this regard, it is extremely important to know which type of miRs can be modulated by nutrients typical of MD, and to evaluate the possible role of nutrient-induced miRs as chemoprevention therapy.

MiRs are also present in all the biological fluids of our body. The possibility to monitor the changes of metabolic miR profiling in the blood stream after prolonged diet intervention in humans could be a relevant achievement. Currently, this topic is in its infancy; in cancer, the study of the association between miRs expression and diet has been carried out mainly using tumor cell lines or animal models. The absorption and metabolism of nutrients at the molecular level have been studied with high-throughput “omics” technologies. The results obtained led to the recognition of certain nutrients able to regulate gene expression, at the base of nutrigenomics [156]. It is expected that miRs expression may also change in response to certain dietary bioactive agents, such as PUFAs, vitamins and phytochemicals and some important data are reviewed as follows: 

#### 2.3.1. Fatty Acids

The development of tumors such as colon cancer [157,158,159,160], breast cancer [161], and glioblastoma [162] is inversely related to the intake of ω-3 PUFA. In contrast, diets rich in ω-6 PUFA (linoleic acid, arachidonic acid and LA, AA) favor both the initiation and promotion of colon cancer [163,164]. In mice, Davidson and colleagues studied the effect of a diet based on corn oil-cellulose compared with a diet based on fish oil (EPA and DHA) and pectin in the presence of carcinogens: their results demonstrated an increased expression of miR-16, miR-19b, miR-21, miR-26b, miR-27b, miR -93, 200c, and miR-203 and the decreased expression of some of their direct targets, such as, PTK2B, TCF4, PDE4B, HDAC4, and IGF1 [158], thus suggesting some different molecular mechanisms involving the fish oil diet. Vinciguerra *et al.* observed that unsaturated fatty acids inhibit PTEN expression in human hepatocytes by up-regulating miR-21 synthesis via mTOR/NF-κB-dependent signaling, exemplifying a regulatory mechanism by which fatty acids affect PTEN expression and trigger liver disorders [165].

Butyrate has chemoprotective properties acting as an inhibitor of histone deacetylase, decreasing proliferation and increasing apoptosis in tumor cells of the colon-rectum [166,167,168,169]. Human colon cancer cells (HCT116) treated with butyrate reduce the expression of different miRs belonging to the miR-17-92-18b, miR-106a and miR-106b-25 clusters [170] mediated by p21, which is a direct target of miR-106b. Experiments in rats showed that butyrate, generated by fermentable fiber and fish oil (EPA and DHA) have a synergistic protective action towards colon tumorigenesis. The increased expression of miR-19b, -26b, -27b, -200c, -203 and the concomitant decrease of their targets expression mediate the tumor suppression mechanisms [171].

#### 2.3.2. Vitamins

All-trans-retinoic acid, the most biologically active metabolite of vitamin A, acts as a tumor suppressor factor in lung, liver, bladder, prostate, breast, and pancreatic cancer models [172]. In breast cancer cells (MCF-7), exposure to retinoic acid inhibited cell proliferation by inducing miR-21 [173].

Recent studies show that vitamin D may exert its protective effects by modulating the expression of miRs and their targets. Vitamin D3 down-regulated miR-181a and miR-181b in human myeloid leukemia cells, resulting in an up-regulation of p27Kip1 and p21Cip1 and cell cycle arrest [174].

#### 2.3.3. Phytochemicals

As described in previous paragraphs, many studies showed that the consumption of foods rich in polyphenols was associated with the prevention of chronic diseases [175,176,177,178]. In particular, quercetin, hesperidin, narangin, anthocyanins, catechins, proanthocyanin, caffeic acid, ferulic acid, and curcumin, act through a common mechanism envisaging the modulation of five miRs, *i.e.*, miR-30c, miR-291b-5p, miR-296-5p, miR -373, and miR-467b [179].

The treatment of human pancreatic cancer cells with curcumin led to a significant up-regulation of eleven miRs and down-regulation of eighteen miRs [180]. Among all, miR-22 was the most significantly up-regulated and was associated with the suppression of Sp1 and estrogen receptor 1, while miR-199a* was the most significantly down-regulated miR. Curcumin and its synthetic analogue, curcumin diflourinated (CDF), alone or in combination, down-regulate miR-200 and miR-21 expression, inducing the up-regulation of its target PTEN in pancreatic cancer cells [181].

Hereafter, changes in blood levels of miRs after the consumption of specific nutrients could be used as biomarkers to monitor the metabolic effects of dietary intervention over time and thus identify dietary interventions that may protect our body from the development of cancer.

## 3. Gut Microbiota, Inflammation and Cancer

Inflammaging and immunosenescence are the main culprits of the changes in microbiota composition in older people [182,183]. It is well known that the human digestive tract is colonized by over 100 trillions of bacteria, which constitute the so-called “gut microbiota” (GM) [184]. These microorganisms, responsible for the degradation of certain complex substances ingested by diet, allowing their digestion and the absorption of certain micronutrients, contribute substantially to our metabolism, and are also essential for the normal development of the immune system. Longitudinal studies have shown that the intestinal microbiota is extremely malleable and could be altered in response to changes in the environment, geography, genetics, metabolism, age, antibiotic treatments, stress, and diet. This plasticity allows the human body to optimize performance while preserving metabolic and immune homestasis as well as health [185].

The impact of the habitual diet on the GM of the elderly [186,187] has been recently highlighted in a study demonstrating the correlation of diet with the inflammatory status, residence and a different rate of health decline upon aging [188]. The balance of carbohydrates, proteins and fats has a profound influence on the maintenance of GM homeostasis [189,190,191]. The adoption of a specific diet, of animal origin, rather than a predominantly vegetable one, determines a different composition of the GM at the expense of interindividual differences in microbial gene expression [192]. Moreover, a diet rich in animal fats induces the development of inflammation and intestinal diseases by modification of the GM.

Our group has shown that centenarians have a different composition of the GM in respect to young subjects and that this is associated with an increase of the “inflammatory state” represented by high levels of pro-inflammatory cytokines (IL-6 and IL-8) [193].

The dysbiosis condition of GM in the elderly can trigger the development of carcinogenic processes in the intestinal mucosa. The number of cases of colorectal cancer (CRC), in fact, increases in the elderly population. The greatest number of CRC occurs in the elderly, with nearly 70% of cases diagnosed in those older than age 65 and 40% diagnosed in those over 75 years of age [194].

It has been demonstrated that mice with a compromised GM homeostasis are prone to developing an inflammatory state in the intestinal mucosa, which, in turn, predisposes them to cancer development [195].

The next generation sequencing techniques allowed, with extreme accuracy, the identification of the composition of GM associated with CRC. The feces of CRC patients compared with those of healthy subjects are particularly rich in pro-inflammatory opportunistic pathogens and microorganisms responsible for metabolic disorders. The “good” GM, with protective function on the mucous membrane, is rather very poor. It is therefore possible to assume that there are “families” of microorganisms able to perform a pro-carcinogenic function (*Fusobacterium*, *Prevotella*, *Coprobacillus*), as well as other families able to perform a protective function of the intestinal mucosa such as *Bifidobacterium* and *Faecalibacterium* [196].

The mechanisms by which microorganisms induce the development of the CRC are varied and include the development of a chronic inflammatory process, the production of toxic metabolites, the development of genotoxins that acts directly on the cell cycle, on the development of DNA and the activation of food heterocyclic amine which are pro-carcinogenic compounds [197].

Among the various metabolites produced by the intestinal microbiota, pro-carcinogenic molecules and molecules with important protective functions have been identified. Secondary bile acids, ammonia, certain amines, phenols and hydrogen sulfide are toxic metabolites, while butyrate plays an anti-proliferative action, energizes the intestinal cells and has shown an apoptotic action against CRC cells *in vitro* [198].

Thus, due to the strong influence of nutrition on GM composition and metabolism [187], the adoption of an anti-inflammatory dietary pattern such as the Mediterranean Diet will contribute to the maintenance of a “good” GM with healthy outcomes.

## 4. A Systems Biology Approach to Diet, Inflammation and Cancer

Systems biology aims to understand how a biological system as a whole responds to internal and external stimuli. Metabolomics [199], instead, aims to measure the global dynamic metabolic response of a living system to biological stimuli or genetic manipulation as a whole, and represents the cutting-edge methodology to fully understand the system-wide effects that diet has on any living organism. The latter approach neatly superimposes to the first: the final result is in both cases a “top-down” view of all the biochemistry processes involved in a complex organism, even if at distinct levels.

Important efforts were made to allow researchers to readily process metabolomics data. The Human Metabolome Project [200] seeks to reproduce what the Human Genome Project did in the genome field, hosting a rapidly growing database of thousands of human metabolites, along with their spectroscopic data. Similarly, the LIPID Metabolites and Pathway Strategy [201] is progressively characterizing human lipids. Metabolomics represents a first-class tool to understand diseases where other approaches are falling short [202]. Metabolome-wide association (MWA) studies, deeply intertwining genome-derived concepts to the “metabolome” world, will in the near future represent a standard approach [203,204,205,206]. A recent study by Watson *et al.* [207] successfully identified metabolites that affect *Caenorhabditis elegans* gene expression and physiology, exploiting an interspecies systems biology approach. Influence of a herring-based diet on sterol metabolism and protein turnover in mice was recently identified, with clear implications also on disease development [208].

The common thread connecting cancer, inflammation and diet is now the focus of metabolomics studies, where the goal is to identify individual metabolites representing end-points of perturbed molecular pathways. These altered molecular pathways may then be further investigated in depth, exploiting other “-omics” techniques [209,210,211]. In a work by Sreekumar *et al.* (2009), the authors were able to successfully identify 87 metabolites distinguishing prostate cancer from benign prostate tissue [212]. More recently, de Boer *et al.* designed a protocol to easily screen the population to detect CRC [213] (or its precursor, advanced adenoma) relying only on volatile organic compound (VOC) analysis. This finding, if confirmed, will pave the way to new, effective and cost-saving methods to perform preventative, large-scale screening, of the population. 

To strengthen the proofs in favour of this inter-systems link, focused cohort-based studies are needed. The European Prospective Investigation into Cancer and Nutrition (EPIC) did exactly that: starting from a cohort of 2380 subjects they analysed a panel of 127 serum metabolites [214], looking for correlations among metabolite networks and different conditions, *i.e.*, physical activity energy expenditure, obesity, and waist circumference, shedding light on the possible adoption of some of the metabolites under analysis as markers to evaluate subjects health. Additionally, an independent cohort study investigating the effect of a diet based on healthy Nordic foods [208], found a lower incidence of colorectal cancer in women following such a diet. The same study also suggests that the Nordic population could better improve their health following diets centered on Nordic food, breaking the dogma that a “Mediterranean Diet” represents the best possible nutritional intervention in all cases, independently from anthropological considerations.

In our bird’s eye voyage through the three different worlds of diet, inflammation and cancer, we showed how these worlds are indeed deeply intertwined. The modulatory effect that unsaturated fatty acids has on the immune systems, the inflammatory response of which can then fuel the micro-environment propelling tumour progression, is just one of the examples of these extensive and branched connections. Any effort to understand the functioning of one of these phenomena must take into consideration all of them, or faces a highly probable failure. In some of the just cited articles, authors fruitfully applied systems biology approaches to unravel the mechanistic reasons underlining the effects they observed. Most of these efforts led to results indicating important advances in the field. In the future, widespread adoption of these analysis techniques will permit breakthrough discoveries that may eventually have a deep impact on public health.

## 5. The Mediterranean Diet and Healthy Aging: the European Project NU-AGE Targeted on Inflammaging to Prevent Age-Related Diseases as a Whole

EU member states are experiencing an extraordinary increase in the life expectancy, which is predicted to reach 84.6 years for women and 82.5 years for men in 2060 [215]. As a direct consequence of the improvement of socio-economic-environmental conditions, the increasing longevity of European citizens will also imply a wide range of societal responsibilities, such as the increased incidence of age-related diseases and the resulting impact on health care cost. Consequently, there is an urgent need to provide policies for preventing aging and related disorders, such as cancer and neurodegenerative disorders among others, allowing the maintenance of reasonably good health as long as possible.

One of the most fascinating anti-aging strategies seems to be the possibility of reducing inflammaging without compromising the physiological role of inflammation, which is essential for survival [5]. At present, nutrition represents the most powerful and flexible tool that we have to reach a chronic and systemic modulation of the aging process in order to improve the health status of the elderly population.

Several studies analized the effect of an individual food or nutrient on a particular form of cancer. However, the overall dietary pattern is more than the sum of the single foods or nutrients eaten and the effect of a dietary intervention on any health outcome is strongly influenced by genetic and environmental factors. Studies regarding the role of the MD on cancer prevention are often conducted analyzing the association between MD adherence and the onset of a specific neoplasia. Studies considering in a comprehensive and integrated way the effect of a balanced whole MD, followed for a consistent period of time, on the chronic and systemic inflammation typical of aging are still scant in the scientific literature.

To rectify this gap in knowledge, the European Project NU-AGE (ClinicalTrials.gov Identifier, NCT01754012) [216,217] will study in a comprehensive and integrated way, the effect of a whole MD newly designed according to the nutritional needs of people over 65 years of age called “NU-AGE diet” [218] on the health status of elderly people in the EU.

The specific rationale of NU-AGE is to enroll in the study free-living, apparently healthy, elderly people including pre-frail subjects (a large segment of the elderly population having the potential to benefit from diet change). All the volunteers will be characterized before and after dietary intervention by measuring a number of robust parameters capable of providing reliable data about different domains/subsystems (health and nutritional status, physical and cognitive functions, immunological, biochemical and metabolic parameters). A sub-group of subjects will be further characterized by advanced techniques (genetics, epigenetics) and highthroughput “omics” (transcriptomics, metagenomics, pyrosequencing, HITChip array) in order to identify cellular and molecular targets and mechanisms responsible for the effects of the whole diet intervention.

The massive amount of data collected from the NU-AGE nutritional intervention will be stored in an ad-hoc built database that, based on an integrated statistical analysis and a system biology approach, will allow the identification of nutritional risk factors in the elderly associated with inflammaging, and to identify the pathways and networks responsive to the NU-AGE diet [219].

This approach will allow an evaluation of the whole-organism response considering several tissues and organs/systems as a functional network instead of assessing the single tissue and organ responses separately, as in previously funded projects, which thereby missed the fundamental cross-talk between tissues and organs/systems. For a detailed description of the entire project it is suggested to read the recently published NU-AGE project dedicated special issue [220].

## 6. Conclusions

Chronic inflammation plays a pivotal role in each stage of carcinogenesis from the initial genetic or epigenetic changes to tumour progression and metastatic diffusion [23,25]. Thus, the chronic, low-level inflammatory state typical in the elderly, that we have named “inflammaging”, likely represents one of the links between aging and cancer.

Inflammaging is both local and systemic, and a variety of organs and systems provide inflammatory stimuli that accumulate lifelong [3]. The key mediators of the inflammatory response activate, in turn, the NF-κB pathway that can be considered the link among cellular senescence, inflammaging and cancer.

A suitable intervention to combat inflammation is a modification of dietary habits. Actually, several studies reported that a healthy lifestyle and a balanced diet might provide benefits to health not only by preventing the risk of diseases but also through facilitating recovery and improving survival. The MD represents one of the best examples of a healthy diet, considered a heritage of humanity by UNESCO, and is considered pivotal in many public health programs in Europe. The acceptance of a Mediterranean lifestyle indeed has a beneficial and preventive role in the onset of cancer and other diseases associated with increased level of inflammation, oxidative damage and angiogenesis.

However, even if the outcomes of the MD on health are well known, knowledge of the reasons of these outcomes is still rather poor. While recent nutrition research focused on the effects of specific dietary constituents, it is still unclear which are the molecular and cellular pathways triggered by the MD as a whole. To this aim, the NU-AGE project is studying in depth the effects of a one-year whole MD in a representative sample of four geographic and cultural areas of Europe (Northern, Eastern, Central and Southern) by measuring an unprecedented number of parameters, including those obtained from “omic” high-throughput analyses, and considering them in a systems biology perspective in order to get a comprehensive view of this dietary intervention on inflammation in old age. This strategy could also hopefully provide insight on diet as cancer prevention; NU-AGE and its study design were noted, in a special issue of the journal *Nature*, devoted to aging studies, as ‘‘the kind of large, longitudinal study that scientists the world over are clamouring for’’ [221].
